# Evaluation of Antibiotic Prescribing Pattern and Appropriateness among Hospitalized Pediatric Patients: Findings from a Malaysian Teaching Hospital

**DOI:** 10.3390/idr14060089

**Published:** 2022-11-17

**Authors:** Muhammad Eid Akkawi, Randa Mahmoud Taffour, Abdulkareem Mohammed AL-Shami

**Affiliations:** 1 Department of Pharmacy Practice, Faculty of Pharmacy, International Islamic University Malaysia, Kuantan 25200, Malaysia; 2 Quality Use of Medicines Research Group, Faculty of Pharmacy, International Islamic University Malaysia, Kuantan 25200, Malaysia; 3 Department of Pediatric Medicine, South Qunfudah Hospital, Al Qunfudah 28821, Saudi Arabia; 4 Faculty of Pharmacy, University College MAIWP International (UCMI), Kuala Lumpur 68100, Malaysia

**Keywords:** antibiotic, pediatric inpatients, inappropriate prescribing, hospital, Malaysia

## Abstract

**Background**: Antibiotics are commonly prescribed for hospitalized children. However, only a limited number of studies have evaluated antibiotic use in this population. The current work assessed the indication, prescribing pattern and appropriateness of antibiotics among pediatric inpatients. **Methods**: A retrospective cross-sectional study was conducted at the pediatric wards of a teaching hospital in Malaysia. Electronic charts of inpatients (≤12 years old) admitted in 2019 were reviewed. Antibiotic indication, selection, dosing regimen, route of administration and duration of treatment were evaluated using the national antibiotic guidelines (NAG). A binomial logistic regression was applied to test potential predictors of inappropriate antibiotic prescribing (IAP) incidence. **Results**: Out of 702 pediatric inpatients, 292 (41.6%) patients were given antibiotics and met the inclusion criteria. More than half of the patients (57.9%) were males, with a median age of 2.5 years. A total of 385 and 285 antibiotics were prescribed during hospitalization and at discharge, respectively. Azithromycin, co-amoxiclav and cefuroxime were the top three prescribed agents. Out of 670 prescriptions, IAP was identified in 187 (28%) prescriptions that were issued for 169 (57.9%) out of the 292 patients included in the study. Improper antibiotic selection, wrong dose and unnecessary antibiotic prescribing accounted for 41%, 34% and 10% of the identified IAP, respectively. Giving lower-than-recommended doses (28%) was more prevalent than prescribing higher doses (5%). The use of two antibiotics and treating upper respiratory tract infections were independent risk factors for IAP incidence. **Conclusions**: Prescribers did not adhere to the NAG in more than one quarter of the prescriptions. This may increase the risk of treatment failure, adverse drug reactions and the development of antibiotic resistance.

## 1. Introduction

Antibiotic resistance is increasingly becoming a global health threat, with the irrational use of antibiotics being the major contributor to the development of antibiotic resistance [1,2]. According to the World Health Organization (WHO), the rational use of antibiotics refers to the use of an antibiotic that is appropriate for a patients’ clinical condition, with doses that meet their individual needs for an adequate period of time [3]. Antibiotic resistance is associated with increased morbidity, mortality and healthcare costs. It is estimated that antibiotic resistance contributes to the death of 23,000 patients and an additional cost of more than 20$ billion per year in the United States [4]. Antibiotics are commonly prescribed for hospitalized pediatric patients. This is because children are more prone than adults to contracting infections that are the leading cause of hospital admission in this population [5,6]. It was reported that at least half of the hospitalized children in developing countries received antibiotic treatment [7,8,9,10]. This high percentage of antibiotic prescribing is almost always associated with different types of inappropriate antibiotic prescribing (IAP), which includes unnecessary antibiotic use as well as incorrect antibiotic selection, dose or duration of treatment [9,11,12]. IAP may result in ineffective treatment, adverse drug reactions (ADRs), prolongation of hospitalization and unnecessary additions to cost [8,13]. 

To avoid IAP, the WHO recommends implementing hospital stewardship programs that include using local guidelines for diagnosis and treatment [1]. Consequently, adhering to local/national antibiotic guidelines is one of the key practices to prevent IAP. Studying the pattern of antibiotic prescribing can help in assessing the prescribers’ adherence to antibiotic guidelines. It was found that respiratory tract infections are the most commonly encountered infections in pediatric wards globally [10,11,14]. Subsequently, penicillins and cephalosporins are the top prescribed classes of antibiotics for children [10,11,14]. However, more than one third of these antibiotics were not appropriately prescribed [11,12]. 

In Malaysia, respiratory tract infections were also reported as the primary driver for caregivers to bring their children to the emergency department [15] and primary care clinics, where more than 35% of the patients received at least one antibiotic [16]. Limited data are available about antibiotic use in pediatric wards in Malaysia. A one-month study that reported the results from a tertiary hospital in Malaysia also found that respiratory tract infections were the leading cause of antibiotic use. However, the appropriateness of the prescribed antibiotics was reported to be 99.8% [17]. It is worth noting here that a review of the Malaysian ADR reporting system revealed that systemic anti-infective agents were the most common therapeutic group reported for ADRs in pediatric patients [18]. To the best of the authors’ knowledge, there are no studies evaluating the appropriateness of antibiotic prescribing among hospitalized pediatric patients in Malaysia. The objective of this study is to assess the indication, prescribing pattern and appropriateness of antibiotics that are prescribed for hospitalized pediatric patients.

## 2. Methods

### 2.1. Study Design and Setting

A retrospective cross-sectional study was conducted at the pediatric wards of a teaching referring hospital in the state of Pahang, Malaysia.

### 2.2. Study Population and Data Collection

Data collection and analysis were conducted in early 2020. Electronic database of pediatric patients (≤12 years old) who were admitted to the pediatric wards during the previous year (January to December 2019) was reviewed. Patients who were prescribed at least one systemic antibiotic and hospitalized for at least 24 h were considered eligible for inclusion in the study. As the study focused on the general pediatric wards, patients who were admitted to the intensive care unit (ICU) were excluded. In addition, because the dosing regimen for patients above 12 years old is usually similar to that used in adults, pediatric patients aged 13–18 years old were also excluded. Patients’ demographic information, medications, past medical history and length of hospitalization were collected.

### 2.3. Measurement

The national antibiotic guidelines (NAG) of 2014 and 2019 [19,20] were used to assess the appropriateness of the prescribed antibiotics. Antibiotic prescribing was deemed inappropriate if the antibiotic was unnecessary prescribed for a viral infection or if the selection, dose, duration of treatment or route of administration did not adhere to the NAG. The selection of antibiotic was considered improper if it was not listed in the NAG as one of the recommended or alternative options for a particular infection. Antibiotic dosage was assessed based on the total daily dose. When an infectious condition was not found in the NAG, Uptodate^®^ was used as a reference. Therefore, the types of IAP investigated in this study were antibiotic without indication, improper antibiotic selection, inappropriate route of administration, low dose, high dose, incorrect duration of treatment and insufficient treatment where two antibiotics are indicated but only one was given.

### 2.4. Sample Size

The site of the study is a referral hospital for the whole state of Pahang. The population of Pahang is estimated to be 1.7 million, and the percentage of pediatrics ≤ 12 years old is 20% [21]. The formula used to calculate the required sample size is as follows [22]:

$$n^{\prime }=\frac{{\mathrm{NZ}}^{2}P\left(1-P\right)}{\left[d^{2}\left(N-1\right)+Z^{2}P\left(1-P\right)\right]}$$

where

n′ = sample size with finite population correction,
N = population size,
Z = Z statistic for a level of confidence, which is 1.96,
P = expected proportion of inappropriate antibiotic use in hospitalized pediatrics, and
d = precision, which is considered as 0.05.

Based on a previous study [23], P was set as 0.2.

$$n^{\prime }=\frac{300,000\times {\left(1.96\right)}^{2}\times 0.2\left(1-0.8\right)}{\left[{\left(0.05\right)}^{2}\times 29,999+{\left(1.96\right)}^{2}\times 0.2\left(1-0.8\right)\right]}=246$$

The minimum required sample size to represent the hospitalized pediatric population in Pahang was 246 patients.

### 2.5. Statistical Analysis

The mean (SD) and median (IQR) were computed for the patients’ characteristics and descriptive results. The data were analyzed using the Statistical Package for the Social Sciences version 24.0 (IBM SPSS Statistics 24). The Shapiro–Wilk normality test was performed to test the normality of continuous variables. The chi-squared test was used to compare the incidence of IAP between subgroups based on the patients’ characteristics. Binomial logistic regression was used to test potential predictors of IAP. Age, gender, history of medical illnesses, duration of hospitalization, type of infection and the number of antibiotics given in the hospital were entered into the regression model. Significance was set at the value of 0.05. 

## 3. Results

### 3.1. Patients’ Characteristics

During the period of the study, 702 pediatric patients were admitted to the hospital. Out of these, 292 patients (41.6%) met the inclusion criteria and had complete information. More than half of the patients (57.9%) were males and about half of the patients (45.2%) have never experienced any medical illness. The age ranged from one month to 12 years old, with a median age of 2.5 years. The majority of the patients (73.6%) were hospitalized for ≥3 days. Table 1 shows the demographic and medical-related information of the patients.

### 3.2. The Pattern and Indications of Antibiotics

**Figure 1:**
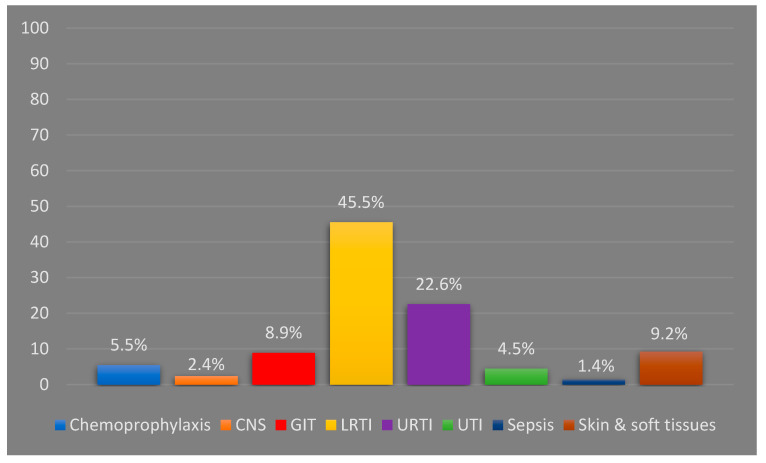
Antibiotic indications based on the body systems (N: 670). CNS: central nervous system; GIT: gastrointestinal tract; LRTI: lower respiratory tract infection; URTI: upper respiratory tract infection; UTI: urinary tract infection.

A total of 385 antibiotics were prescribed for the patients during hospitalization. About one quarter of the patients (28.4%) were prescribed with more than one antibiotic. Most of the patients (83.9%) were discharged with at least one antibiotic, and the majority of them were prescribed with the same antibiotic(s) given during hospitalization. The total number of antibiotics prescribed at discharge was 285 agents (Table 2). The mean ± SD duration of antibiotic use was 6.79 ± 3 days. Lower respiratory tract infections (LRTIs) led to the hospitalization of 135 patients (Figure 1), with pneumonia being the most reported diagnosis (Table 1). Meningitis was the least encountered infection, reported in five patients only. 

Table 2 shows the pattern of antibiotic prescription for the involved patients. Macrolides were the most frequently prescribed antibiotic class (107 prescriptions in the ward; 90 prescriptions at discharge) followed by a combination of penicillins and beta-lactamase inhibitors (102 prescriptions in the ward; 79 prescriptions at discharge) and second-generation cephalosporines (88 prescriptions in the ward; 80 prescriptions at discharge). Azithromycin, co-amoxiclav and cefuroxime were the top three prescribed agents. Several antibiotics like cefepime, clindamycin and meropenem were prescribed only once. 

### 3.3. Inappropriate Antibiotic Prescribing

**Figure 2:**
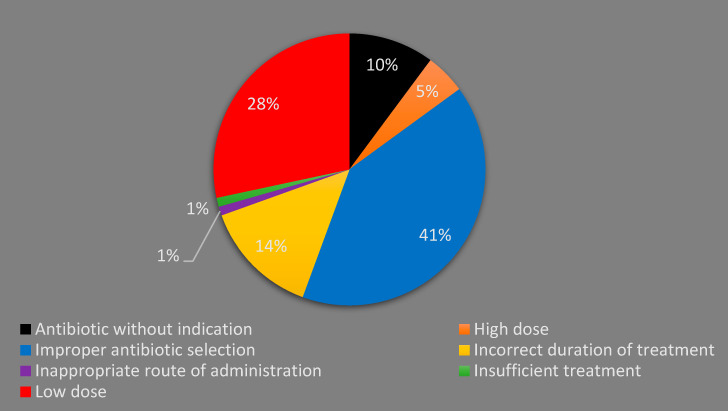
Types of inappropriate antibiotic prescribing (N: 187).

Out of 670 prescriptions, IAP was identified in 187 (27.9%) prescriptions that were issued for 169 patients (57.9%). A few patients (4.8%) were found to have two types of IAP. Improper antibiotic selection (41%) was the most commonly identified IAP. Wrong dosing accounted for 34% of the total identified IAP, and giving lower-than-recommended doses (28%) was far more common than prescribing higher doses (5%). Unnecessary antibiotics were prescribed for 19 patients (6.5%), representing (10%) of the total identified IAP (Figure 2).

No significant difference was found in the incidence of IAP between male and female patients (chi-square test, *p* = 0.664) or different age categories (chi-square test, *p* = 0.292). However, the incidence of IAP was associated with the type of infection (Fisher’s exact test, *p* = 0.002) and the number of antibiotics used in the ward (Fisher’s exact test, *p* = 0.043). 

To control for other variables such as the history of medical illnesses and the duration of hospitalization, a binomial logistic regression was applied, and the incidence of IAP was considered as the variable of interest. Age, gender, history of medical illnesses, duration of hospitalization, type of infection and the number of antibiotics given in the hospital were entered into the regression model. Female gender, using one antibiotic and not having a history of medical illnesses were considered as the references for gender, number of antibiotics used and the history of having illnesses, respectively. Age categories and the type of infections were treated as dummy variables, with age < 1 year and chemoprophylaxis being the references. All assumptions required to apply the binomial regression were met before running the model. The model statistically and significantly predicted the incidence of IAP; χ^2^ = 36.05, *p* = 0.002. 

As shown in Table 3, the model revealed that the type of infection and the number of antibiotics used were significant predictors of the incidence of IAP (Table 3). The use of two antibiotics was associated with 1.86 times higher possibility to have IAP compared with using one antibiotic. In addition, the use of antibiotics to treat URTIs had a 7.15-fold higher possibility of having IAP than the use of antibiotics for chemoprophylaxis.

## 4. Discussion

The literature is lacking in studies evaluating the use of antibiotics in pediatric inpatients. This study assessed the pattern and appropriateness of antibiotics prescribed for hospitalized children in a Malaysian teaching hospital. It was found that 292 out of 702 (41.6%) hospitalized pediatric patients were prescribed with at least one systemic antibiotic. The antibiotic prescribing rate of the current study is relatively lower than that reported from other developing countries such as Turkey (54.6%) [11], Gambia (54.1%) [10], Nigeria (63%) [8], Costa Rica (65%) [24] and India (66%) [25]. No similar study was reported from Malaysia. However, the prescribing rate was comparable (43.5%) to what was reported in children brought to the emergency department of a tertiary Malaysian hospital [15]. The majority of the patients (71%) received one antibiotic, which resembles what has been reported from India (71%) [26] and Nigeria (63%) [8]. The median duration of treatment was seven days, which is similar to the findings elsewhere [10,26]. 

About two thirds of the patients (68.1%) were diagnosed with respiratory infections, with LRTIs being more prevalent than URTIs. Pneumonia was the most common type of LRTI, while tonsillitis was the most reported URTI. Acute respiratory infections are the leading cause of children’s hospitalization worldwide, including Malaysia [10,11,14,17,27,28]. This is because children have an immature immune system and are usually surrounded by peers who could carry infections [12]. The main approach to treat pneumonia was a monotherapy of co-amoxiclav, cefuroxime or azithromycin or a combination of co-amoxiclav/cefuroxime and azithromycin. Additionally, co-amoxiclav monotherapy was the mainstay treatment used for tonsillitis/pharyngitis. Because of this, azithromycin, co-amoxiclav and cefuroxime were the top three prescribed antibiotics in this study. Penicillins and cephalosporines were repeatedly reported in the literature as the most common prescribed antibiotic classes for hospitalized children [11,12,14,17,26,28]. However, penicillins without a β-lactamase inhibitor and third-generation cephalosporines, namely ceftriaxone, were the predominately prescribed agents [11,12,14,17,26,28]. The difference in the results between our study and the other mentioned studies can be explained by the preference of the prescribers, where they preferred cefuroxime over ceftriaxone for pneumonia and azithromycin over amoxicillin for tonsillitis/pharyngitis, although these agents are considered as the second-line treatment according to the Malaysian NAG [20]. Third-generation cephalosporines were also commonly used inappropriately in those studies. In addition, co-amoxiclav, which was frequently prescribed in our study for patients with tonsillitis/pharyngitis, was used inappropriately, as will be discussed below. Notably, the use of macrolides in our study was far higher than what is reported elsewhere [11,12,14,26,28]. This can be attributed to the recommendations of the NAG, where azithromycin is recommended as a monotherapy for pneumonia suspected to be caused by atypical bacteria, and it is also the alternative therapy for tonsilitis/pharyngitis. Aminoglycosides (AMGs) use was prevalent in Turkey (16.6%) [11], Nigeria (25.4%) [8] and India (20%) [26]. Nevertheless, they were prescribed for only eight patients (2.73%) with sepsis or a urinary tract infection (UTI) in our study. Again, the difference can be explained partly by the NAG’s recommendations, as AMGs are not recommended for respiratory infections. This claim is supported by a similar result reported from another Malaysian study, where AMGs were used in 2.9% of the cases [17]. Additionally, sepsis and UTIs were more prevalent in those studies than ours [11]. 

Inappropriate antibiotic prescribing was found in 57% of the patients. This incidence rate of IAP is higher than what was found in Turkey (47%) [11], Pakistan (41%) [12] and Ethiopia (28%) [29]. This could be attributed to the lack of established hospital antibiotic guidelines in our teaching hospital and the difference in the types of infections encountered. On the other hand, non-adherence to NAG recommendations was found to be higher among pediatric outpatients in Malaysia (71.8%) [16]. Improper antibiotic selection represents 42% of all types of IAP identified. This was mainly attributed to the prescribing of co-amoxiclav for URTIs despite not being recommended by the NAG. Co-amoxiclav was also inappropriately selected as a perioperative prophylaxis. Unnecessary antibiotic use (antibiotic without indication) was identified in 19 prescriptions, where antibiotics were prescribed for acute gastroenteritis (AGE) and bronchiolitis. However, both conditions are believed to be viral infections that do not need empirical antibiotic use [20,30]. The use of antibiotics for viral infections and AGE was also reported as a common IAP in other countries [9,29]. It is important to note here that no laboratory confirmatory test for diagnosis was reported in the patients’ electronic charts. Thus, unnecessary antibiotic use could be underestimated in our study. This may explain the differences in the results of this study and studies from other countries where IAP was much higher [12]. However, most of the studies did not differentiate between the wrong selection of antibiotic and unnecessary antibiotic prescribing and considered them to be the same [11,28]. 

One third of the identified IAP in the current study was related to wrong daily dosage. In most of these cases, low doses were used. The use of lower-than-recommended doses were mainly associated with the use of cefuroxime for pneumonia. This finding is in consistent with a previous study conducted in a general pediatric ward in Malaysia, where incorrect dosage represented 31% of the overall identified medication errors [31]. In fact, the scenario was worse among Malaysian pediatric outpatients where wrong antibiotic dosing was identified in 64% of the prescriptions [16]. One possible reason for inappropriate dosing is the inadequate dosing information for pediatric patients that is available in the most commonly used drug reference in Malaysia: the Monthly Index of Medical Specialties (MIMS) [32]. Likewise, inappropriate dosage was also reported in the literature as one of the top encountered types of IAP in pediatric patients [12,28,29], with underdose being the predominant dosing error [12,29]. The duration of treatment was inappropriate in 26 (9%) patients. In 19 out of those 26 patients, the physicians prescribed antibiotics for a shorter-than-recommended duration, which theoretically might lead to a failure of treatment and/or an increase in bacterial resistance. However, growing data show no evidence of these claimed negative outcomes [33,34]. Studies from Pakistan and Ethiopia showed a higher rate of prescribing antibiotics with the wrong duration of treatment [12,29].

Incidence of IAP was not associated with the patients’ demographic characteristics or the length of hospital stay. These findings are not consistent with other studies, where advanced age of children was reported by Yehualaw et al. as an independent factor for IAP [29]. However, that study involved children ≤ 18 years old. Iftikhar et al. found that long length of hospital stay was correlated with antibiotic errors in hospitalized children [12]. Nevertheless, that study evaluated antibiotics prescribed for respiratory infections only. We found that the number of antibiotics and the type of infection were significantly associated with the incidence of IAP. The applied logistic regression revealed that prescribing two antibiotics and treatment of URTIs were independent factors for IAP. It is logical that prescribing multiple medications increases the possibility of medication errors compared with monotherapy, and this was supported by findings from pediatric wards [35]. On the other hand, the most common types of IAP in this study were related to antibiotics prescribed for URTIs, which clearly explains why treating URTIs was an independent risk factor for IAP. 

## 5. Limitations

This study was conducted in a single hospital, which limits the generalizability of the findings to other pediatric wards in Malaysia. The data are also lacking diagnostic confirmatory tests for the infections. Thus, unnecessary antibiotic use could be underestimated in our study. Additionally, the study is limited by its retrospective design, which does not allow us to discuss the prescribed regimens, which could have been appropriate in some cases, with their prescribers. 

## 6. Conclusions

This study showed that antibiotics are commonly prescribed for hospitalized pediatric patients, and LRTI is the leading cause of using antibiotics. IAP was identified in more than half of the cases. The most common types of IAP were improper drug selection, incorrect dosing, and incorrect duration of treatment. This indicates that prescribers at the study site did not strictly adhere to the Malaysian NAG. Nonadherence to the guidelines may increase the risk of treatment failure, adverse drug reactions, and the development of antibiotic resistance.

## Figures and Tables

**Table 1:** Patients’ demographic and medical-related information (N: 292).

Variable	N (%) *
Gender	
Male	169 (57.9)
Female	123 (42.1)
Age (year)	
<1	55 (18.8)
1–3	118 (40.4)
3–6	62 (21.2)
>6 years	57 (19.5)
Mean ± SD	3.4 ± 2.9
Median (range)	2.5 (0.1–12)
Duration of hospitalization (days)	
Mean ± SD	3.5 ± 1.96
Median (range)	3.0 (1–13)
Having a history of medical illness(es)	
Yes	132 (45.2)
No	160 (54.8)
The most common diagnosis	
Pneumonia	120 (41.1)
Tonsilitis	30 (10.3)
Pharyngitis	21 (7.2)
Gastroenteritis	12 (4.1)
Urinary tract infection	10 (3.4)
Number of antibiotics used in the ward	
1	209 (71.6)
2	74 (25.3)
3	9 (3.1)
Discharged with antibiotic	
Yes	245 (83.9)
No	47 (16.1)
Total ** duration of antibiotic treatment (days)	
Mean ± SD	6.79 ± 3
Median (range)	7 (1–30)
Having IAP	
No IAP	123 (42.1)
One type of IAP	155 (53.1)
Two types of IAP	14 (4.8)

* Unless otherwise stated. ** Inward plus discharge. IAP: inappropriate antibiotic prescribing.

**Table 2:** The pattern of antibiotic prescription during hospitalization and at discharge.

Antibiotic Class	Antibiotic Agent	N (%) In-Ward	N (%) At Discharge
Penicillins	Amoxicillin	3 (0.78)	2 (0.70)
	Ampicillin	15 (3.90)	11 (3.87)
	Cloxacillin	18 (4.68)	14 (4.93)
	Benzylpenicillin	7 (1.82)	1 (0.35)
	Phenoxymethylpenicillin	4 (1.04)	5 (1.75)
Penicillins + beta-lactamase inhibitor	Amoxicillin + clavulanic acid	98 (25.45)	79 (27.82)
	Piperacillin + tazobactam	2 (0.52)	-
Macrolide	Azithromycin	102 (26.49)	84 (29.58)
	Clarithromycin	3 (0.78)	3 (1.06)
	Erythromycin	2 (0.52)	3 (1.06)
Second gen. cephalosporines	Cefuroxime	88 (22.86)	80 (28.07)
Third gen. cephalosporines	Ceftriaxone	20 (5.19)	-
	Ceftazidime	1 (0.26)	-
	Cefotaxime	2 (0.52)	-
Fourth gen. cephalosporines	Cefepime	1 (0.26)	1 (0.35)
Aminoglycosides	Gentamicin	7 (1.82)	1 (0.35)
	Amikacin	1 (0.26)	-
Carbapenems	Carbapenem	1 (0.26)	-
	Meropenem	1 (0.26)	-
Sulfonamide	Sulfamethoxazole + trimethoprim	2 (0.52)	1 (0.35)
Glycopeptides	Vancomycin	2 (0.52)	-
Lincosamides	Clindamycin	1 (0.26)	-
Imidazole derivatives	Metronidazole	4 (1.04)	
Total		385 (100)	285 (100)

**Table 3:** Variables included in the binomial regression models to predict inappropriate antibiotic prescribing.

Variable	Unstandardized Coefficient (B)	*p* Value	OR	95% CI for OR	
Gender (Male)	0.004	0.986	1.005	0.598	1.687
Age categories		0.275			
1–3 years	−0.349	0.343	0.705	0.343	1.450
3–6 years	−0.800	0.054	0.449	0.199	1.015
>6 years	−0.479	0.262	0.619	0.268	1.432
Having a history of medical illness(es)	0.092	0.728	1.096	0.654	1.836
Days of hospitalization	0.040	0.582	1.041	0.903	1.199
Type of the infection		0.002			
CNS	0.382	0.696	1.466	0.215	9.980
GIT	0.831	0.219	2.295	0.610	8.631
LRTI	0.493	0.389	1.638	0.533	5.038
Sepsis	−0.909	0.483	0.403	0.032	5.122
Skin and soft tissues	0.262	0.692	1.299	0.355	4.758
URTI	1.968	0.002	7.158	2.081	24.624
UTI	1.226	0.127	3.406	0.704	16.475
Number of AB used		0.040			
Two antibiotics	0.620	0.047	1.859	1.009	3.425
Three antibiotics	1.949	0.079	7.023	0.797	61.911

AB: antibiotic; OR: odds ratio; CI: confidence interval.

## Data Availability

Data are available upon a request from the corresponding authors.
